# Investigation of Internalized Weight-Related Stigma: Progression to Dietary Addiction and the Role of Stress

**DOI:** 10.7759/cureus.55007

**Published:** 2024-02-26

**Authors:** Athina Papatsaraki, Despoina Pappa, Alexandra Koreli, Freideriki-Eleni Kourti, Panagiota Manthou, Konstantina Chasaki, Ioannis Koutelekos, Nikoletta Margari, Maria Theodoratou, Chrysoula Dafogianni

**Affiliations:** 1 Nutrition, Hellenic Open University, Patras, GRC; 2 Nursing, University of West Attica, Athens, GRC; 3 Internal Medicine, Lefkada General Hospital, Lefkas, GRC; 4 Critical Care Medicine, University of West Attica, Athens, GRC; 5 Nursing, Lefkada General Hospital, Lefkas, GRC; 6 Psychology, Hellenic Open University, Patras, GRC

**Keywords:** weight-related stigma, mental well-being, stress, food addiction, internalized

## Abstract

Introduction: Internalizing weight stigma can lead to the development of dietary addiction, as individuals seek food as a coping mechanism for the emotional distress caused by stigma. The influence of stress exacerbates this dynamic, encouraging the reliance on food as a stress-coping strategy.

Methods: Electronic questionnaires were completed in a special electronic form through an online platform. The Two-Factor Weight Bias Internalization Scale (WBIS-2F), the Yale Food Addiction Scale (YFAS), the Depression Anxiety Stress Scale-21 (DASS-21), Positive and Negative Affect Schedule (PANAS), the Life Orientation Test (LOT), the Brief Resilience Scale (BRS) were used at this study to examine the prevalence of Internal Weight Stigma (IWS) among adults in Greece, evaluate the degrees of stress/anxiety, food addiction, mental resilience, emotions, and positive life perspectives within this group, and explore the correlations between stress/anxiety, mental resilience, optimistic life attitudes, and both IWS and food addiction.

Results: 376 participants completed the questionnaire. The average BMI of the participants was 26.3 kg/m2 (SD = 5.9 kg/m2). Almost half of the participants (46.8%) fell within the normal weight range (18.5 ≤ BMI ≤ 24.9), while 28.2% were classified as overweight and 21.0% as obese. Interestingly, a significant majority (63.1%) perceived themselves as heavier than the normal weight range suggests. Most participants demonstrated typical levels of depression, anxiety, and stress, with percentages of 67.3%, 64.9%, and 71.3%, respectively. Resilience exhibited positive associations with optimism and positive emotions while displaying negative connections with depression, anxiety, stress, and negative emotions. Additionally, individuals with greater optimism reported fewer symptoms of despair, anxiety, and stress.

Conclusion: The research highlights the intricate dimensions of mental well-being, emphasizing the need for a holistic comprehension encompassing demographic, psychological, and societal factors. The results indicate potential strategies for intervention to boost resilience, and optimism, and tackle issues such as food addiction, underscoring the significance of fostering a positive body image and self-esteem.

## Introduction

Weight-related stigma is a significant social issue characterized by its pervasiveness and influence on health status. Coping with weight stigma can hinder an individual's psychosocial well-being, potentially resulting in a saddened emotional state, heightened metabolic risk factors, and diminished self-esteem [1]. People with excess weight are often seen as weak-minded, greedy, and unethical. Individuals coping with weight stigma experience discrimination in various areas of their daily lives, including the working environment, education, healthcare system, media, and relationships. This unfavorable social setting can be associated with depressive symptoms, feelings of powerlessness, loneliness, and poor overall psychological functioning among obese people [2]. Consequently, those suffering from weight stigma tend to limit their world to avoid being the object of disdain, putting them at risk of internalizing weight bias [2-3].

Internalizing weight stigma involves adopting negative societal beliefs about body weight, a phenomenon applicable to individuals of any body weight, gender, age, sex, race, or ethnicity. Weight bias internalization, closely linked to self-esteem, provides a precise measure of an individual's thoughts primarily tied to weight stereotypes. Studies indicate that internalized bias is more prevalent in certain groups, specifically among individuals with higher body weight, women, and those who are white, as opposed to black individuals [4]. Additionally, individuals who engage in binge eating exhibit a higher level of internalized bias compared to their non-binge eating counterparts [4-5].

Moreover, researchers have shown that this type of weight stigma is related to adverse psychological symptoms, including decreased quality of life, depression, anxiety, low self-esteem, and poor body image. Physiologically, the risk for metabolic syndrome is higher, and triglycerides are increased [6]. Behavioral issues like disordered eating behaviors are also frequent, and exercise avoidance is noticed [7]. Self-directed weight stigma is connected not just with obesity and food addiction but also with stress and mental resilience. Depression and anxiety, the two most commonly reported types of psychological discomfort in the weight-related stigma mechanism, are strongly linked to food addiction. Previous research indicates that psychological distress caused by weight-related stigma predicts the development of food addiction. Furthermore, it was proposed that fearing being stigmatized by others, rather than self-devaluation when it comes to weight, contributes to the development of food addiction [8].

Beyond negative attitudes, eating disorders, and reduced quality of life, there's limited research on well-being practices and protective factors shaping alternative psychosocial profiles. Specifically, it's been found that overweight individuals seem less happy [9], and subjective well-being (SWB) is well known to have an inverse connection with BMI [10]. Also, the link between obesity and diminished happiness has a more pronounced impact on women than men [11].

Aim

The study aimed to explore the occurrence of Internal Weight Stigma (IWS) in the adult Greek population, assess levels of stress/anxiety, food addiction, mental resilience, emotions, and optimistic life attitudes in this demographic, and investigate the associations of stress/anxiety, mental resilience, and optimistic life attitudes with IWS and food addiction.

## Materials and methods

Methods 

Research data was collected through an electronic questionnaire posted on the institution's website in a special electronic form (Microsoft Forms). This particular method of data collection is user-friendly, cost-effective, and bypasses geographical limitations as it can be done easily and quickly from any part of the country. The questionnaire was completed by the participants anonymously and voluntarily. Participants were advised to share the questionnaire with other potential interested parties after submitting their answers. The sampling of the study was accomplished with the snowball method and 376 people agreed to take part in the study after adequate completion. The sample size of the study was determined by the number of participants at the conclusion of the data collection. The collection of responses took place from November 2022 to July 2023. The study inclusion criteria for the participants were to be adults with a fluent command of the Greek language.

Procedure and ethical considerations

Questionnaires were completed in a special electronic form (Microsoft Form) through an online platform that controls personal data such as internet protocol (IP) addresses. According to the General Data Protection Regulation (GDPR), a privacy and security law enacted by the European Union (EU), the members of the research team declare that they will not have access to the IP addresses of the participants. No personal data was included in the questionnaire. Permission for this study was obtained by the Research Committee of Hellenic Open University, Patras (No. 115014/19-10-2022).

In addition, any data on the participants was removed before statistical analysis was performed. Participants in the study have been properly informed about the purpose and content of the study before consenting to their participation. Before starting the completion process, participants had the mandatory box check (''tick'') whether they agreed to participate in the study or not. If not, they did not have the right to continue. No data was exposed to others apart from researchers and only for the study purposes. The answers were strictly confidential, and only the research team had access to them for research purposes.

Study instruments

The first part of the study questionnaire consisted of consent or non-participation in the research that had to be answered before completing the questionnaire. The following sections included sections on demographic and anthropometric data and six validated measurement instruments: Two-Factor Weight Bias Internalization Scale (WBIS-2F) [12], Yale Food Addiction Scale (YFAS) [13], Depression, Anxiety, and Stress Scale (DASS-21) [14], Positive and Negative Affect Scale (PANAS) [15], the Life Orientation Test (LOT-R) [16], and the Brief Resilience Scale (BRS) [17]. Permissions for use were obtained from the developers of all scales.

Statistical analysis

The statistical analysis was carried out using the statistical software program Statistical Package for Social Sciences (SPSS), version 26.0 (IBM Corp., Armonk, NY), with the Kolmogorov-Smirnov test used to examine the distribution of quantitative variables. The nonparametric Mann-Whitney and Kruskal-Wallis tests were employed to compare quantitative variables between two or more groups. The Bonferroni adjustment was used to correct for type I errors caused by repeated comparisons. Spearman's correlation coefficient was utilized to determine the link between the two quantitative variables. A hierarchical linear regression analysis was used to find independent components linked with the WBIS-2F scale dimensions, as well as to calculate the dependency coefficient and standard error.

Participants' demographics and somatometric data were entered using the enter technique, while the resilience, optimism, stress, sadness, anxiety, food addiction, and positive and negative emotion measures were entered sequentially. Logarithmic transformations were used to perform the linear regression analysis, as the distributions of the WBIS-2F scale dimensions showed skewness. Finally, to check the internal reliability of the investigated scales, the Cronbach's α coefficient was used. Significance levels are two-sided, and statistical significance was set at 0.05.

## Results

The study achieved a response rate of 97.4%, with 376 out of 386 participants agreeing to and successfully completing the questionnaire. Participants' mean age was 33.6 years (SD=12.0). Most of the participants were single (53.5%, n=201). Private employees were at 27.1% (n=102), students at 60.4% (n=117), and individuals with under- or post-graduate/Ph.D. university programs at 60.4% (n=227) (Table 1).

**Table 1:** Demographic characteristics of participants

		n	%
Gender	Male	68	18.1%
	Female	308	81.9%
Age (years), mean (SD)		33.6 (12)	
Marital status	Single	201	53.5%
	Married	148	39.4%
	Divorced	24	6.4%
	Widowed	3	0.8%
Profession (or professional occupation)	State employee	92	24.5%
	Private employee	102	27.1%
	Free license employee	31	8.2%
	Unemployed	18	4.8%
	Student	117	31.1%
	Household	9	2.4%
	Pensioner	4	1.1%
	Farmer	3	0.8%
Educational level	Primary school graduate	2	0.5%
	High school	27	7.2%
	University graduate	55	14.6%
	2-year college graduate	3	0.8%
	Postgraduate	56	14.9%
	PhD	5	1.3%
	College	1	0.3%
	Student (undergraduate/postgraduate/PhD)	227	60.4%
If student, which is your educational Institution?	Hellenic Open University	78	20.7%
	University of West Attica	62	16.5%
	University of Patra	26	6.9%
	International University of Greece	16	4.3%
	College graduate	14	3.7%
	University of Crete	6	1.6%
	University of West Macedonia	4	1.1%
	National and Kapodistrian University of Athens	3	0.8%
	Hellenic Mediterranean University	3	0.8%
	University of Thessaly	3	0.8%
	University of Ioannina	2	0.5%
	University of Peloponnese	2	0.5%
	Other universities	8	2.4%

The participants’ BMI mean was of 26.3 kg/m2 (SD=5.9 kg/m2). 46.8% (n=176) of participants had normal weight (18.5≤ BMI≤24.9). 28.2% (n=106) were overweight and 21.0% (n=79) were obese. However, the majority (63.1%, n=236) considered themselves heavier than normal.

Emotion and state of mind scales

The majority of participants showed normal levels of depression, anxiety, and stress at 67.3%, 64.9%, and 71.3% respectively (Table 2). The reliability coefficient in all scales was acceptable (>0.7).

**Table 2:** Description of participants’ depression; anxiety and stress levels on the DASS-21 scale DASS-21: Depression Anxiety Stress Scale-21

		n	%
DASS-21 Depression	Normal	253	67.3%
	Mild	20	5.3%
	Moderate	59	15.7%
	Severe	13	3.5%
	Extremely severe	31	8.2%
DASS-21 Anxiety	Normal	244	64.9%
	Mild	21	5.6%
	Moderate	50	13.3%
	Severe	22	5.9%
	Extremely severe	39	10.4%
DASS-21 Stress	Normal	268	71.3%
	Mild	34	9.0%
	Moderate	26	6.9%
	Severe	29	7.7%
	Extremely severe	19	5.1%

All emotional and mental state scales have been proven to be significantly correlated. Resilience is linked favorably with optimism and positive effect, but negatively with depression, anxiety, stress, and negative effect. Furthermore, those with higher levels of optimism reported lower symptoms of despair, anxiety, and stress. Participants who experienced higher levels of depression, anxiety, and stress reported less positive and more negative feelings (Table 3).

**Table 3:** Spearman’s correlation between Dass-21, LOT, BRS, and PANAS scales BRS: Brief Resilience Scale, DASS-21: Depression Anxiety Stress Scale-21, LOT: Life Orientation Test, PANAS: Positive and Negative Affect Schedule

		LOT	DASS-21 Depression	DASS-21 Anxiety	DASS-21 Stress	DASS-21 Overall	PANAS Negative Affect Score	PANAS Positive Affect Score
Brief Resilience Scale (BRS)	rho	0.53	-0.42	-0.36	-0.37	-0.41	-0.37	0.38
	P	<0.001	<0.001	<0.001	<0.001	<0.001	<0.001	<0.001
Life Orientation Test (LOT)	rho	1.00	-0.46	-0.36	-0.41	-0.44	-0.38	0.38
	P		<0.001	<0.001	<0.001	<0.001	<0.001	<0.001
DASS-21 Depression	rho		1.00	0.69	0.79	0.91	0.64	-0.32
	P			<0.001	<0.001	<0.001	<0.001	<0.001
DASS-21 Anxiety	rho			1.00	0.71	0.85	0.57	-0.24
	P				<0.001	<0.001	<0.001	<0.001
DASS-21 Stress	rho				1.00	0.93	0.65	-0.22
	P					<0.001	<0.001	<0.001
DASS-21 Overall	rho					1.00	0.69	-0.29
	P						<0.001	<0.001
PANAS Negative Affect Score	rho						1.00	-0.11
	P							0.031

Yale Food Addiction Scale (YFAS)

Chocolate (47.3%), fried potatoes(31.4%), fried food (30.3%), chips (27.7%), and pizza (27.4%) were the most commonly reported foods that created problems for participants. The YFAS scores ranged from 0 to 7 (a higher score indicates greater food reliance), with a mean range of 2.62 (SD=1.55), with a respectable 0.7 dependability coefficient. 17.8% of the sample showed evidence of food dependence (YFAS score ≥3). The YFAS score showed a substantial correlation with all emotional and mental state categories (Table 4). Individuals who scored highly on resilience, optimism, and good effect also showed a decreased need for food. Conversely, it was shown that individuals with elevated levels of anxiety, stress, sadness, and negative affect were more reliant on food.

**Table 4:** Correlation between WBIS-2F, and BRS, LOT, DASS-21, PANAS, and YFAS BRS: Brief Resilience Scale, DASS-21: Depression Anxiety Stress Scale-21, LOT: Life Orientation Test, PANAS: Positive and Negative Affect Schedule, WBIS-2F: Two-Factor Weight Bias Internalization Scale, YFAS: Yale Food Addiction Scale

		Self-devaluation WBIS-2F	Weight-related distress WBIS-2F
BRS	rho	-0.12	-0.15
	P	0.024	0.003
LOT	rho	-0.13	-0.19
	P	0.009	<0.001
DASS-21 Depression	rho	0.12	0.32
	P	0.018	<0.001
DASS-21 Anxiety	rho	0.15	0.20
	P	0.005	<0.001
DASS-21 Stress	rho	0.08	0.26
	P	0.113	<0.001
Overall DASS-21	rho	0.11	0.29
	P	0.033	<0.001
YFAS	rho	0.11	0.34
	P	0.038	<0.001
PANAS Negative Affect Score	rho	0.16	0.29
	P	0.002	<0.001
PANAS Positive Affect Score	rho	-0.12	-0.13
	P	0.020	0.009

WBIS-2F's self-devaluation dimension ranged from 1 to 5, with a mean of 2.17 (SD=0.86); the "weight-related distress" dimension ranged from 1 to 6.6, with a mean of 3.26 (SD=1.02). The reliability coefficients in both dimensions were 0.7, which is considered satisfactory. Resilience, optimism, and positive affect all had a favorable relationship with self-deprecation and weight-related suffering. Participants with higher levels of depression, anxiety, and stress had higher WBIS-2F scores across both domains. The WBIS-2F scale was found to have a positive connection with negative impacts and food dependence (Table 5).

**Table 5:** Spearman’s correlation coefficients between YFAS and DASS-21, BRS, LOT, and PANAS scales BRS: Brief Resilience Scale, DASS-21: Depression Anxiety Stress Scale-21, LOT: Life Orientation Test, PANAS: Positive and Negative Affect Schedule, YFAS: Yale Food Addiction Scale

		Yale Food Addiction Scale (YFAS)
BRS	rho	-0.13
	P	0.012
LOT	rho	-0.15
	P	0.004
DASS-21 Depression	rho	0.43
	P	<0.001
DASS-21 Anxiety	rho	0.38
	P	<0.001
DASS-21 Stress	rho	0.42
	P	<0.001
DASS-21 Overall	rho	0.45
	P	<0.001
PANAS Negative Affect Score	rho	0.31
	P	<0.001
PANAS Positive Affect Score	rho	-0.20
	P	<0.001

The "self-devaluation" dimension revealed that married people had considerably lower scores (less self-depreciation) than singles, with median values of 2 (1.33 ─ 2.67) and 2.17 (1.5 ─ 2.83), respectively, when linked with gender, BMI, family status, and education. Likewise, in terms of "self-devaluation," those who believe their weight is normal had a considerably lower score than those who believe their weight is abnormal: median 2 (1.33 ─ 2.67) vs. 2.17 (1.67 ─ 2.83), p = 0.002(p>0.05). In multivariate hierarchical linear regression with the "self-devaluation" dimension as a dependent variable, only those who consider themselves heavier than normal showed an important correlation (step, a0.019). In the second phase, the result remained significant (p=0.012). Moreover, the negative effect was positively linked with self-depreciation (p<0.001). No significant correlation was found between age, gender, BMI, marital status, or education (p>0.05). It was observed, after Bonferroni correction, that participants with underweight/normal BMI had significantly less weight-related distress in comparison with obese participants (p=0.007) (Table 6).

**Table 6:** Correlation of “weight-related distress” with participants’ characteristics + Mann-Whitney test, ++ Kruskal-Wallis test WBIS-2F: Two-Factor Weight Bias Internalization Scale

		Weight-related distress WBIS-2F		P
		Mean (SD)	Median (IQR)	
Gender	Male	3.07 (0.92)	3.14 (2.14 - 3.64)	0.104+
	Female	3.3 (1.04)	3.21 (2.57 - 4)	
BMI	Underweight/normal	3.17 (0.99)	3.14 (2.43 -3.86)	0.019++
	Overweight	3.2 (1.01)	3 (2.43 - 3.86)	
	Obese	3.56 (1.08)	3.43 (2.86 - 4.29)	
Married	No	3.31 (1.05)	3.29 (2.57 - 4)	0.168+
	Yes	3.17 (0.98)	3.14 (2.43 - 3.86)	
Working	No	3.18 (1.01)	3 (2.43 - 3.86)	0.194+
	Yes	3.31 (1.03)	3.29 (2.57 - 4)	
Level of education	Primary education/high school graduate	3.23 (1.06)	3.14 (2.43 - 4)	0.860++
	College graduate	3.28 (1.03)	3.29 (2.43 - 4)	
	University graduate	3.14 (0.96)	3.14 (2.43 - 3.86)	
	Post-graduate/PhD	3.28 (1.05)	3.14 (2.57 - 3.86)	
Do you consider yourself heavier than normal?	No	3.13 (0.93)	3.14 (2.43 - 3.86)	0.132+
	Yes	3.34 (1.07)	3.29 (2.43 - 4.07)	

Age was not shown to be significantly related to the "weight-related distress" factor. Conversely, there was a positive correlation (p = 0.020) between the last subjects of BMI. In the multivariate hierarchical linear regression using the "weight-related distress" dimension as the dependent variable, only BMI showed a statistically significant correlation. In the first phase, overweight people reported less weight-related distress than obese participants; in the second step, this finding was no longer significant (due to the correlation with the YFAS scale). A positive association was established between the negative impact of food dependence and weight-related distress (Table 7).

**Table 7:** Multivariate hierarchical linear regression with dependent varia “weight-related distress” dimension. + dependence factor, ++ coefficient standard error PANAS: Positive and Negative Affect Schedule

		β+	SE++	P
1ο step	Sex (female vs male)	0.033	0.020	0.102
	Overweight vs Obese	-0.047	0.021	0.028
2ο step	Sex (female vs male)	0.017	0.019	0.359
	Yale Food Addiction Scale (YFAS)	0.022	0.005	<0.001
	PANAS Negative Affect Score	0.004	0.001	<0.001

## Discussion

The study reveals a significant gender imbalance, with women accounting for 81.9% of the participants. However, this result did not seem to affect the study findings. The group, primarily young people aged 33.6 years and pursuing higher education, is well-educated and aware of health-related issues. The majority of participants are single, possibly influenced by their younger age and marital status. The study also shows that despite half of the participants having a normal BMI, a significant majority perceive themselves as heavier than normal, potentially impacting their emotional well-being.

The DASS-21 scale reveals that most people experience sadness, anxiety, and stress. Resilience and optimism can improve emotional well-being, while those with more conditions experience more negative emotions. A study using data from various scales found a positive correlation between resilience and reduced depression, anxiety, and stress levels. The LOT also showed strong negative correlations with negative emotional states, suggesting a positive outlook protects against mental distress. However, 17.8% of the sample demonstrated food addiction, with chocolate, fried potatoes, and pizza being the most problematic foods. YFAS scores significantly correlated with emotional and mental scales, suggesting greater food addiction is associated with poorer emotional states. Participants with higher YFAS scores showed higher levels of negative affect (rho = 0.31, p < 0.001) and lower levels of positive affect (rho = -0.20, p < 0.001).

The WBIS-2F found average scores for self-devaluation and weight-related distress, with acceptable reliability coefficients of 0.7 for body image self-perception. The study suggests that weight-related distress is not universal, but a proportion of the population experiences it significantly. Positive attributes like resilience and optimism predict positive self-views, but can also correlate with self-devaluation and weight-related distress. Higher resilience and optimism are linked to more positive self-perceptions. Negative emotions may worsen body image and eating behaviors. Marital status was found to be a significant factor, with married participants reporting less self-devaluation than singles. Self-perception also plays a significant role in psychological health.

In a multivariate hierarchical linear regression analysis, the self-evaluation dimension was significantly linked to participants' perception of being overweight. This effect remained significant even after controlling for negative effects, which were also positively correlated with self-deprecation. No significant correlations were found between age, gender, BMI, marital status, and education levels. Our study's findings on the relationship between BMI and weight-related distress are consistent with existing research. Studies have consistently shown an association between higher BMI and increased mental health concerns [18], including weight-related self-stigma depression and psychological distress [19].

While some research reports no association between stress and BMI, others have found that stress is associated with higher BMI. The results of this study confirm the complex relationship between BMIs and mental health, which is in line with similar studies [20]. Additionally, the analysis revealed that individuals with a BMI categorized as underweight or normal experienced significantly less weight-related distress compared to obese participants. This suggests that BMI categories significantly influence how individuals experience and internalize weight-related distress.

The study highlights the importance of a holistic approach to mental health and attitudes towards body weight, considering personal resilience, emotional states, and social perceptions. It found a significant negative correlation between weight perception and BMI, suggesting that overweight individuals reported more distress than those classified as obese. The YFAS scores suggest that food dependence may have a more significant impact on weight-related distress than BMI categories alone. Higher YFAS scores are more likely to experience greater weight-related distress due to addictive eating behaviors and loss of control over food consumption. The PANAS Negative Affect Score also plays a role in predicting weight-related distress. Marital status was found to be a significant factor, with married participants reporting less self-devaluation than singles [21,22].

Namely, the analysis revealed a strong positive correlation between weight-related distress and negative effect, as measured by the PANAS Negative Affect Score. This finding supports the idea that individuals who experience higher levels of negative emotions are more likely to report distress related to their weight. Our research presents a clear understanding of the emotional implications of obesity highlighting a positive correlation between weight-related distress and negative affect. Pasco et al. also observed that increasing BMI correlates with heightened negative affect among women, leading to increased feelings of distress and other negative emotions [23]. Both sets of findings support the idea that weight distress is closely associated with negative emotional states, highlighting the psychological aspect of obesity.

The YFAS and PANAS Negative Affect Score are significant predictors of weight-related distress, with the YFAS linked to increased psychopathology and the PANAS Negative Affect Score strongly associated with adverse weight-related outcomes. The study suggests that gender may not be a significant predictor of weight-related distress, as psychological factors like body dissatisfaction and eating disorder symptoms may mediate the relationship [24,25].

Puhl and Heuer's research suggests that the stigma associated with being overweight can be profound, leading to significant psychological distress among overweight individuals. This may explain the increased distress among those on the threshold of obesity, as being on the threshold may be associated with increased anxiety and stigma. The findings emphasize the importance of considering psychological factors in weight-related distress and challenge the field to broaden its understanding of weight status perception and experience [26].

The study emphasizes the positive impact of optimism and resilience on mental health, highlighting their role in enhancing emotional well-being and reducing issues like depression and anxiety. It also highlights the interplay between optimism and resilience, with optimism aiding motivational coping and resilience mediating its effects [27-30].

Limitations

While the present research provides valuable insights into the connection between internalized weight-related stigma, dietary addiction progression, and stress, several limitations should be acknowledged. Firstly, the study's cross-sectional design restricts the establishment of causal relationships among variables, emphasizing the need for longitudinal research to better delineate temporal associations. Additionally, the reliance on self-report measures introduces the possibility of response bias and may not fully capture the nuanced and complex nature of participants' experiences. The generalizability of the findings may also be limited due to the specific demographic characteristics of the sample. The majority of the participants were female and highly educated. This lack of diversity in the sample may limit the applicability of the findings to other demographic groups. Furthermore, the study primarily focuses on psychological factors, and the potential influence of biological or genetic components on dietary addiction remains unexplored. Future research should aim to address these limitations by employing diverse methodologies, considering broader demographic groups, and incorporating interdisciplinary approaches to provide a more comprehensive understanding of the intricate interplay between internalized weight-related stigma, dietary behaviors, and stress.

## Conclusions

The study reveals a gender imbalance and educational influence, with a predominantly female population. It highlights the complex relationship between perceived and actual weight status, impacting body image and psychological well-being. Resilience and optimism play protective roles, while food addiction affects emotional states. Self-devaluation and weight-related distress vary, with married individuals reporting less self-devaluation. The results of the research and their possible utilization in creating interventions to enhance resilience, optimism, and a positive body image have the potential to address concerns like food addiction and promote overall mental well-being on a larger scale.
